# COVID-19: laboratory diagnosis for clinicians. An updating article

**DOI:** 10.1590/1516-3180.2020.0240.14052020

**Published:** 2020-06-22

**Authors:** Luisane Maria Falci Vieira, Eduardo Emery, Adagmar Andriolo

**Affiliations:** I MD. Clinical Pathologist, Regional President of the Sociedade Brasileira de Patologia Clínica/Medicina Laboratorial (SBPC/ML), Rio de Janeiro (RJ), Brazil; Technical Director of the Laboratório Lustosa, Belo Horizonte (MG), Brazil.; II MD, MSc. Clinical Pathologist, Coordinator of the Immunology Committee of the Sociedade Brasileira de Patologia Clínica/Medicina Laboratorial (SBPC/ML), Rio de Janeiro (RJ), Brazil; Member of the Technical Chamber of Clinical Pathology/Laboratory Medicine, Regional Council of Medicine of the State of Rio de Janeiro, Rio de Janeiro (RJ), Brazil.; III MD, PhD. Clinical Pathologist, Associate Professor and Full Professor at Escola Paulista de Medicina - Universidade Federal de São Paulo (EPM-UNIFESP), São Paulo (SP), Brazil; Head of the Discipline of Clinical Medicine and Laboratory Medicine at EPM-UNIFESP, São Paulo (SP), Brazil; Editor of the Jornal Brasileiro de Patologia e Medicina Laboratorial, Rio de Janeiro (RJ), Brazil.

**Keywords:** Coronavirus, Clinical laboratory techniques, Pandemics, Molecular biology, Biological process, Laboratory diagnosis, Viral infection

## Abstract

COVID-19 (coronavirus disease 2019) is an infectious disease caused by the new coronavirus associated with severe acute respiratory syndrome 2 (SARS-CoV-2). Coronaviridae comprises a large family, of which at least seven members are known to cause respiratory diseases in humans. Coronaviruses have the ability to infect virtually all major groups of animals and, eventually, can infect humans. SARS-CoV-2 is the third coronavirus to cross the species barrier and infect humans. This virus was identified in an outbreak of pneumonia cases in Wuhan city, Hubei province, China, in December 2019. Its entire genome is inscribed on a single strand of ribonucleic acid. Some proteins present on the surface of the virus act as facilitators for its entry into host cells, while others, apparently, are related to its pathogenesis. Coronaviruses are responsible for respiratory infections in humans and some animals. The infection is often mild to moderate in intensity, but some coronaviruses may cause serious illnesses, such as severe acute respiratory syndrome (SARS), which occurred in 2002, and the Middle East respiratory syndrome (MERS). Coronaviruses can activate an excessive and unregulated immune response, which may promote SARS development. Although the lungs are one of the target organs, the hypoxia mechanism is systemic and other organs begin to suffer both through lack of oxygen and through deregulation of inflammation control mechanisms.

## INTRODUCTION

COVID-19 (coronavirus disease 2019) is an infectious disease caused by the new coronavirus associated with severe acute respiratory syndrome 2 (SARS-CoV-2). Coronaviridae comprises a large family, and at least seven coronaviruses are well known for causing respiratory diseases in humans. Coronaviruses have the ability to infect virtually all major groups of animals, among which some host other species that may infect humans. The current understanding is that SARS-CoV-2 is the third zoonotic coronavirus to have crossed the barrier between species and become capable of infecting humans over the past two decades.

## THE CORONAVIRUS THAT CAUSES COVID-19

SARS-CoV-2 is a new betacoronavirus belonging to the large viral family of Coronaviridae, first identified in an outbreak of pneumonia cases in Wuhan city, Hubei province, China, in December 2019. The name COVID-19 was chosen as the name for this new infection as an acronym of coronavirus disease 2019, i.e. from corona “co”, virus “vi”, disease “d” and the number 19 indicating the year of its appearance. Efforts are being made by the World Health Organization (WHO) to ensure that the nomenclature of viruses and their infections no longer refers to geographical locations, as it did traditionally, in order to combat the stigma resulting from this practice.

The entire SARS-CoV-2 genome is inscribed on a single strand of RNA (ribonucleic acid). This type of virus undergoes genetic mutations more frequently than DNA (deoxyribonucleic acid) viruses, given that RNA viruses have less ability to correct any transcription errors.^1^ SARS-CoV-2, in particular, is a single-stranded RNA virus that is capable of synthesizing about 29 different proteins. Some of these proteins are present on the surface of the virus and act as facilitators of its entry into host cells, while others, apparently, are related to its pathogenesis.

Characteristically, coronaviruses are responsible for respiratory infections in humans and some animals. Most of the time, the infections caused by viruses in this family are mild to moderate in intensity and manifest as common colds. Some coronaviruses can cause more serious illnesses, such as severe acute respiratory syndrome (SARS), which occurred in 2002, and the Middle East respiratory syndrome (MERS), which still occurs in a well-defined region.

Coronaviruses can activate an excessive and unregulated immune response that is harmful to the host. These responses can contribute to the development of SARS. Autopsies on patients with COVID-19 worsened by SARS have revealed hyperactivation of effector T cells (CD8+) with high concentrations of cytotoxic granules. Reports describing the immune profile of patients critically ill with COVID-19 have suggested that hyperactivation of the cellular immune pathway may be a mediator of respiratory failure, shock and multiple organ failure.

SARS-CoV-2 binds to the human host cell via the ACE2 receptor (angiotensin-converting enzyme 2), and its input mechanism depends on sequential action by the serine protease TMPRSS2 enzyme. These data suggest that several therapeutic targets are possible, including the interleukin (IL)-6-STAT3 axis, which is associated with cytokine release syndrome (CRS).

Another structural protein of the virus is thought to have the ability to displace the iron that is present in hemoglobin. This would reduce the oxygen transportation capacity and provide the low level of saturation that is observed in some of the patients who evolve poorly. Additionally, release of iron ions in high quantities would cause oxidative damage, thus triggering an intense inflammatory process that might result in the condition known as a cytokine storm. Although the lungs are one of the target organs, the hypoxia mechanism is systemic and other organs start to suffer, both through lack of oxygen and through deregulation of the inflammation control mechanisms. Two other organs strongly affected are the liver and kidneys.

The clinical manifestations of COVID-19 can range from none, i.e. a totally asymptomatic state (which may be the case in up to 89% of individuals who become infected), to a situation characterized by mild to critical and fatal symptoms. The symptoms can develop between 2 to 14 days after exposure to the virus,^2^ with an average incubation period of 5.1 days. Hence, the recommended quarantine period is usually 14 days.

Although the proportions may vary in the different populations affected, data provided by the Chinese Center for Disease Control and Prevention (CCDC) have shown that 81% of the patients had mild clinical manifestations; 14% of them had severe manifestations, including hypoxia; 5% of the cases were critical, with respiratory failure, multiple organ dysfunction and shock; and 2.3% of the cases were fatal.^3^

The course of the disease can last for around 16 days after a short incubation period, in mild to moderate cases; or can last for up to 10 weeks if there is a longer incubation period and a severe or fatal outcome. The following clinical scenarios have been estimated from various publications.

- Incubation period of 2 to 14 days (average 5 to 6 days) after infection
- Mild cases: duration of two weeks
- Severe and recovered cases: duration of three to six weeks

A person can be a transmitter even before symptoms appear, and can continue to be a transmitter until they disappear. The peak period for transmission is around five days after the onset of symptoms. It should be noted that because the pandemic is still new, the information provided here may change.^4^

## LABORATORY DIAGNOSIS

When an infected person exhales droplets containing SARS-CoV-2 virions and they are inhaled by someone else, these droplets lodge on the nasal mucosa, which is formed by cells covered with ACE2 receptors. The virus binds with these receptors and thus is able to penetrate and hijack cell machinery in order to produce other virions and infect new cells. While viral multiplication is occurring, this person eliminates virions in large quantities, especially in the first week. This period can be asymptomatic or paucisymptomatic, with fever, dry cough, sore throat, anosmia, ageusia and severe headaches or body pain.^5^ If the immune system is unable to stop the infection at this point, the virus progresses through the respiratory tract to the pulmonary alveoli, which are rich in ACE2 receptors. In the alveoli, leukocytes migrate due to the action of cytokines, which results in disruption of gas exchange, with pneumonia, characterized by productive cough, fever and dyspnea.^6^

In outpatients with flu-like symptoms, the chemosensory disorder causes loss of the sense of the smell and taste that is strongly associated with SARS-CoV-2 infection. This sign should be considered suggestive for clinical screening. Most people thus affected recover this function within weeks, in parallel with resolution of other symptoms.^7^

At this point, some patients’ condition abruptly deteriorates, with development of SARS. Breathing becomes more difficult and oxygenation levels decline. Examinations on plain radiography and computed tomography of the chest reveal the typical ground-glass opacity. Patients need artificial ventilation and many die at this stage of the disease. Patients with severe COVID-19 conditions often develop acute hypoxemic respiratory failure and pneumonia, and 17% to 29% of them develop SARS. However, SARS in these cases differs in several aspects from the usual form of SARS, in which lung compliance is decreased. In patients with COVID-19, lung compliance is high.

The virus, probably with help from the immune response to it, can cause damage to the following other organs:

- Liver: about half of hospitalized patients show signs of liver changes.
- Kidneys: kidney damage, including kidney failure and the need for dialysis, is common in severe cases.
- Intestines: there may be a clinical presentation characterized by gastrointestinal symptoms, especially vomiting, diarrhea and abdominal pain. A “stomach” pain that may be associated with inflammation of the base of the lungs and diaphragm has been described. The lower gastrointestinal tract is rich in ACE2 receptors and about 20% of patients report diarrhea.
- Central nervous system: there have been reports of stroke, possibly due to formation of microthrombi, occurrences of seizures and cognitive changes.
- Eyes: in severe cases, occurrence of conjunctivitis has been described.
- Heart: an increase in cardiovascular events, notably acute myocardial infarction and thromboembolic events, and even disseminated intravascular coagulation, has been described.

## MOLECULAR TESTS FOR SARS-COV-2: REAL-TIME REVERSE TRANSCRIPTION-POLYMERASE CHAIN REACTION (RRT-PCR)

The real-time reverse transcription-polymerase chain reaction with amplification (real-time RT-PCR with quantification, i.e. qRT-PCR) is the methodology that best applies for detection of the SARS-CoV-2 virus. With this technique, it is possible to identify viral RNA.

The genes considered for identification include N, E, S and RdRP. The international protocol developed by the Charité/Berlin Institute^8^ and recommended by the Pan-American Health Organization (PAHO/WHO) has been used by most countries. Initially, laboratory confirmation depended on detection of two genetic markers but, considering the current high rate of virus circulation, confirmation can be given through detection of a single genetic marker.

The recommendation for laboratory confirmation of cases is that two different genetic markers should be detected (for example, the E gene followed by the RdRP gene, as previously described for the Charité protocol). Once circulation of the virus has become established and disseminated in a certain area or country, it would no longer be necessary to perform PCR for both genes, and confirmation would become possible through detection of a single genetic marker. The E gene has slightly higher sensitivity than the RdRP gene, so the Ministry of Health suggests prioritizing the E gene as the marker of choice.^8^

To improve the responsiveness of the public network, rapid molecular tests that can be processed on the automated GeneXpert platform (Cepheid) may be made available in Brazil. This is the same platform as used in the rapid test network for tuberculosis. This test performs qualitative in vitro detection of SARS-CoV-2 nucleic acid through automated real-time PCR, targeting the E and N2 genes, in nasopharyngeal swabs or nasal aspirate/wash specimens from suspected cases of COVID-19.^8^

The SARS-CoV-2 genome was quickly sequenced and was found to show 80% similarity to the SARS-CoV genome and 50% similarity to the MERS-CoV genome. The molecular laboratory diagnosis of COVID-19, in its initial phase, can be made from material collected from the upper respiratory tract (nasopharynx or oropharynx) or from the lower respiratory tract (sputum, tracheal aspirate or bronchoalveolar lavage) and is based on detection of specific viral nucleic acid. Because SARS-CoV-2 is an RNA virus, its identification requires generation of a complementary DNA strand (cDNA), which is obtained through the action of the reverse transcriptase enzyme. After reverse transcription, two primers that promote amplification of two genetic targets are inserted. With a complementary probe, it is possible to observe the molecular content corresponding to that of the target infectious agent.

Important barriers impeding widespread use of RT-PCR for detection of the new coronavirus in Brazil currently exist. Notably, the quantity of test kits and equipment available in this country is insufficient. Moreover, this methodology is laborious when the extraction and testing are manual, which has given rise to a large number of tests pending, mainly, but not exclusively, within the public network of the Central Public Health Laboratories (Laboratórios Centrais de Saúde Pública, LACENs). Nonetheless, it is likely that this scenario will return to normal, considering that production of this test is being greatly stimulated.

This laboratory test is very specific. However, its sensitivity can vary, mainly due to pre-analytical variables such as:

- Stage of infection and viral load in secretions and excretions, mainly with regard to upper respiratory tract samples collected less than three days and more than ten days since the beginning of the contamination;
- Place of collection: It is known that materials from the lower respiratory tract (sputum or bronchoalveolar lavage) tend to be more positive than those from the upper respiratory tract (combined nasal and oropharyngeal swabs);
- Collection, transportation and storage techniques used for samples until their analysis, to avoid degradation of the RNA present in the specimen.

RT-PCR directly detects the presence of specific components of the virus genome. Therefore, it should be used to diagnose the disease in the asymptomatic or pre-symptomatic phase, or in the symptomatic phase within the first 12 days after the onset of symptoms. There are not enough data in the Brazilian literature yet to have an accurate picture regarding the sensitivity and specificity of this methodology in this country. For reference only, the following sensitivity rates can be stated (true positive results in the presence of the disease): 93% for investigations on bronchoalveolar lavage, 72% on sputum, 63% on nasal material and 32% on oropharyngeal material. Detection of the virus in blood, feces, urine and saliva is also possible, but use of these samples for routine diagnosis has not yet been developed. Many of the virus detection studies were carried out only through viewing the virus through electron microscopy and virus neutralization techniques.

RT-PCR for diagnosing SAR-CoV-2 is considered highly specific, and a positive result confirms the presence of the infection (“gold standard”). However, because of the aforementioned problems regarding sensitivity, negative results do not rule its presence out, and it may be necessary to repeat the test on another sample after a few days.

In order to ensure that the tests that are requested are performed, it is essential that the medical order is clear and objective. It is recommended that the requesting physician should indicate the material that is to be collected (for example, secretions from the oropharynx or nasopharynx, sputum, tracheal aspirate or bronchoalveolar lavage) and explain which test is to be performed, i.e. RT-PCR for SARS-CoV-2.

## MOLECULAR PANELS FOR OTHER RESPIRATORY VIRUSES

According to guidance from the Ministry of Health,^8^ patients with SARS who present a negative molecular test for COVID-19 need to undergo testing for other respiratory viruses, including influenza.

Respiratory viruses are the pathogens most frequently associated with acute respiratory infections (ARI), with high morbidity and mortality in children in the first year of life and in immunocompromised adults and the elderly.

Pneumonia is the most common cause of fatal outcomes. Clarifying the viral cause avoids undue administration of antimicrobials and allows identification of the causal virus, thus making it possible to study outbreaks.

For this purpose, samples of respiratory tract secretions can be collected to be subjected to molecular diagnostic tests for detection of specific nucleic acids of various respiratory viruses. These multiplexed molecular panels are popularly known as “viromes”. Their scope may vary between laboratories, but they generally have the ability to detect influenza, parainfluenza, coronavirus, respiratory syncytial virus, adenovirus, metapneumovirus, enterovirus, bocavirus and rhinovirus.

A Chinese study reported that coinfection with other respiratory viruses would be rare.^9^ Another study on the incidence of coinfection showed that molecular detection of a pathogen other than SARS-CoV-2 was not sufficient to ensure absence of infection with the new coronavirus. These results do not support routine use of virome panels, given their low effectiveness in this context. However, one possible exception could be the use of neuraminidase inhibitors in patients infected or coinfected by influenza.^10^

Considering that viral coinfections are relatively frequent, according to the guide of the Influenza Surveillance Laboratory Network of Brazil,^11^ all patients presenting suspected influenza-like illness (ILI) or SARS should be tested for the COVID-19 virus and other respiratory pathogens, using usual laboratory procedures.

## IMMUNOLOGICAL LABORATORY TESTS

In the light of the WHO guidance on the need for mass testing of populations, given the expansion of the pandemic and the current barriers to carrying out RT-PCR at rates compatible with demand, companies producing laboratory diagnostic reagents have started to develop tests for investigating antibodies and antigens related to the virus. The vast existing literature highlights that coronaviruses are immunogenic infectious agents, capable of generating humoral and cellular immune responses in the host.

Just like in all other viral infections, the body reacts to the presence of this virus by producing antibodies, initially those of the immunoglobulin A (IgA) and immunoglobulin M (IgM) classes, and subsequently those of the immunoglobulin G (IgG) class. Presence of specific antibodies against antigenic determinants of SARS-CoV-2 indicates that there was a previous infection, but considering that this is an infectious agent that was only introduced into the community very recently, occurrence of cross-reactions with other coronaviruses currently in community circulation cannot be ruled out. Such occurrences could compromise the specificity of the tests.

Some time is needed for production of these antibodies. On average, the time required is 7 to 10 days after the onset of symptoms for the IgM class antibodies, and 10 days or more for IgG. These numbers clearly indicate that early detection of antibodies is possible , but only in a limited number of patients. As the days go by, the concentrations of both antibody classes increase, and the chance of false-negative results decreases. While the search for viral particles is carried out mainly using secretions and washings, the search for and quantification of antibodies can be carried out using capillary blood, whole blood, serum or plasma. Thus, blood collection is required, either from a fingertip for the rapid test, or from a vein to obtain whole blood.

Several immunological tests are already available on the market, certified by the Brazilian Health Regulatory Agency (Agência Nacional de Vigilância Sanitária, ANVISA), for investigations on IgA, IgM and IgG antibodies and viral antigens. Several methodologies are available:

Automated methodologies: These include the enzyme-linked immunosorbent assay (ELISA), chemiluminescence and electrochemiluminescence. Investigation and quantification of antibodies using these methods are carried out on whole blood, serum or plasma, in a laboratory environment, using analytical equipment that is capable of quantifying antibody levels and performing tests on paired samples, 28 days apart. In general, these methods are more sensitive and specific and less dependent on the operator.

Methodologies for “rapid tests”, more appropriately called “point-of-care testing” (POCT): These consist mainly of immunochromatography. They involve investigation of antibodies in whole blood, serum or plasma using manual methods, which are performed quickly on individual devices and provide results within 10 to 30 minutes. At present, some tests of this type are reported to present performance deficiencies. These tests are still at the stage of evaluation and validation, at both public and private clinical laboratories.

Some rapid tests that are available for investigation of viral antigens in material collected from the nostrils and throat have not yet been studied regarding their effectiveness in relation to molecular tests.

The different performance qualities of the rapid tests that are currently available arise from a variety of technical issues, such as differences in the purification processes used for coronavirus viral antigens (S and N). The aims in these processes are to avoid loss of their three-dimensional format and thus to adequately recognize antibodies (i.e. to maintain antigen quality), achieve the ideal degree of surface sensitization and ensure the quality of reactants, storage and transportation, among other matters.

The literature relating to antibody production in response to antigen stimulus shows that this is an individual response. Therefore, the number of antibodies formed may vary and, consequently, the time of antibody detection may differ, although the vast majority of studies on coronavirus infections have indicated that these are already evident by the seventh or eighth day. Thus, it has been emphasized that patients may take shorter or longer times to manifest the infection. It has been concluded from these studies that awareness of the possible variability of the immunological window period is needed (i.e. the time that elapses between contamination and laboratory detection of antibodies).

It is not yet possible to say with certainty whether the antibodies thus formed are an effective defense against possible reinfection, i.e. whether they confer immunity and, if so, how long they last. It is also not yet possible to clearly state what the role of rapid serological tests for making individual diagnoses is, considering that non-reactive results do not rule out the possibility of SARS-CoV-2 infection. On the other hand, reactive results can lead to a false sense of security with regard to re-exposure to the virus. However, within the context of public health, the greatest utility of serological tests may lie in their use in population-based surveys.

In early April, the WHO recommended that rapid tests should be used for epidemiological purposes only and contraindicated their use for diagnosis. In cases in which RT-PCR tests are non-reactive but COVID-19 infection is suspected, serological tests performed on sequential samples can assist in clarifying the diagnosis.^12^

## SEROPOSITIVITY CERTIFICATE

At the present time, the best definition for an “immunity passport”, would perhaps be that this is a “seropositivity certificate”. There is strong pressure for social life to return to the old “normal” pattern, i.e. for there to be a loosening of social distancing. With the recent development of laboratory tests that detect the presence of antibodies, an expectation has been created that people in whom antibodies against SARS-CoV-2 are potentially detected could be released to resume their usual activities. Studies in this direction are being carried out in some countries of the European Union, especially Germany, and in China and the United States. At the moment, the following elements need to be considered:

- Antibodies start to be detected only one to two weeks after infection;
- It is not yet known whether antibodies detectable through current tests are capable of conferring long-lasting immunity (i.e. whether they would be protective antibodies), or for how long;
- The new tests have been released in a speeded-up manner and still need to be carefully validated.

In the case of SARS in 2002, antibodies were present for two to three years and in the case of MERS, for one year. In the case of COVID-19, this information would be important for making it possible to determine, for example, the retest interval that would be required in order to enable certification of immunity on an ongoing basis and to organize vaccination programs.

It is not certain that the appearance of antibodies indicates that the person is no longer a transmitter or that he/she becomes immune, and for how long. Some people have been shown to carry the virus for a long time (a few weeks).

There are ethical issues. Even if the percentage of false-reactants is not large, non-immune people would be considered immune and exposed, and susceptible people could be discriminated against in the job market. And lastly, it is necessary to avoid enabling trade in this type of certificate, including through fraud.

## LABORATORY EVALUATION OF THE INFLAMMATORY STORM

Cytokines are low molecular weight proteins that are released by various types of cells, especially those that make up the immune system, and have a role in intercellular signaling. Among various actions, the cytokines released by the cells of the immune system act to modulate the inflammatory response, from the time when the organism is attacked by infectious agents. The term “cytokine” is derived from two Greek words “cyto” for cell, and “kinos” for movement. The rationale for this nomenclature, which emphasizes the mobile nature of these cells, is based on the fact that, because they are small molecules, they have great mobility in body fluids and have the ability to recruit different cells of the immune system to act together.

The inflammatory response is an extremely complex event, involving numerous cellular and humoral agents. Among other purposes, it seeks to identify, isolate, neutralize and eliminate agents that are harmful to the body. It involves participation of cellular and humoral elements that act in a coordinated manner, with self-modulation of their intensity of response to an invading agent. However, dysregulation of the inflammatory response may sometimes occur. In these situations, excessive quantities of cytokines are released, and these activate and recruit cells of the immune system such that an oversized response is generated, thus resulting in hyperinflammation. This condition is called a “cytokine storm” and results in generation of a microenvironment that is harmful to the organism itself.

The cytokine storm concept apparently began to be studied in the early 1990s. It was correlated with several viral infections and was recognized during the SARS outbreak in 2002, as a factor presenting a high risk of mortality. The term was only coined in 2005, when avian influenza caused by the H5N1 virus occurred, and its occurrence was linked to a high mortality rate due to an exacerbated pro-inflammatory response. In some non-infectious diseases, such as multiple sclerosis, cytokine storms can also be observed. Since then, cytokine storms have been described in relation to several respiratory diseases caused by viruses of the coronavirus family, such as MERS in 2012 and, more recently, SARS-CoV-2.^13^ The main cause of death in cases of COVID-19 is respiratory failure caused by SARS.^14^

Another syndrome related to the immune response is secondary hemophagocytic lymphohistiocytosis (sHLH), a little-known condition that is characterized by fulminant hypercytokinemia and rapidly evolves to multiple organ failure. It has been described in about 3.7% to 4.3% of sepsis cases,^15^ but it is more often described in viral infections.^16^ Clinically, it is characterized by the presence of constant fever, cytopenia and high levels of ferritin, interleukins (IL), granulocyte-colony stimulating factor (GCSF), interferon-γ inducible protein 10, monocyte chemoattractant protein*-*1, macrophage inflammatory protein 1-α and tumor necrosis factor-α.^17^

SARS occurs in about 50% of patients with sHLH.^18^^,^^19^ Comparison of groups of patients with SARS in association with the new coronavirus via laboratory results has shown significant differences between survivors and non-survivors. The most striking differential parameters include leukocyte counts, absolute lymphocyte and platelet counts and the concentrations of albumin, total bilirubin, serum urea, creatinine, myoglobin, cardiac troponin, C-reactive protein (CRP) and interleukin-6 (IL-6).

In patients with COVID-19, ferritin and IL-6 levels have been shown to be good predictors of fatality, as shown in Table 1, adapted from the work by Ruan et al.^14^ This emphasizes the hypothesis of the presence of a hyperinflammatory response.

**Table 1.** Laboratory parameters among patients with COVID-19, as modified by Rual et al.^14^

Laboratory parameters	Reference range	Non-survivors mean (SD)	Survivors mean (SD)	P-value
Time between symptom onset and collection, in days		11.6 (6.8)	9.8 (4.3)	0.07
Leukocytes, × 10^9^/l	3.50-9.50	10.62 (4.76)	6.76 (3.49)	< 0.001
Lymphocytes, × 10^9^/l	1.10-3.20	0.60 (0.32)	1.42 (2.14)	< 0.001
Hemoglobin, g/l	130.0-175.0	127.0 (16.7)	127.6 (16.3)	0.82
Platelets, × 10^9^/l	125.0-350.0	173.6 (67.7)	222.1 (78.0)	< 0.001
Serum albumin, mg/dl	3.50-5.20	2.88 (0.38)	3.27 (0.38)	< 0.001
Serum alanine aminotransferase, U/l	9.0-50.0	170.8 (991.6)	48.68 (83.1)	0.35
Serum aspartate aminotransferase, U/l	15.0-40.0	288.9 (1875.5)	40.7 (57.8)	0.31
Total serum bilirubin, mg/dl	0.3-1.5	1.1 (0.63)	0.75 (0.40)	0.001
Serum creatine, mg/dl	0.50-1.50	1.03 (0.64)	0.81 (0.27)	0.02
Serum creatine kinase, U/l	50.0-310.0	319.4 (838.5)	231.7 (862.3)	0.56
Serum lactic dehydrogenase, U/l	120.0-250.0	905.8 (2619.1)	297.9 (110.4)	0.08
Serum cardiac troponin, pg/ml	2.0-28.0	30.3 (151.0)	3.5 (6.2)	< 0.001
Serum myoglobin, ng/ml	0.0-146.9	258.9 (307.6)	77.7 (136.1)	< 0.001
Serum C-reactive protein, mg/l	0.0-5.0	126.6 (106.3)	34.1 (54.5)	< 0.001
Serum interleukin-6, pg/ml	0.0-7.0	11.4 (8.5)	6.8 (3.61)	< 0.001
Serum ferritin, ng/ml	21.8-274.7	1297.6 (1030.9)	614.0 (752.2)	< 0.001

SD = standard deviation.

## OTHER LABORATORY PARAMETERS

COVID-19 is a systemic infection with significant impacts on the hematopoietic system and hemostasis. Lymphopenia can be considered to be a cardinal laboratory sign, and is potentially prognostic. The neutrophil/lymphocyte and peak platelet/lymphocyte ratios can help to assess the severity of cases. Over the course of the disease, a longitudinal assessment of the dynamics of lymphocyte counts and inflammatory indices (including lactic dehydrogenase, CRP and IL-6) can help to identify cases with a worse prognosis and indicate the need for more aggressive interventions. Other biomarkers, such as procalcitonin and ferritin, are also being considered as factors that indicate worse prognosis.

Hypercoagulability is common in patients who are hospitalized due to COVID-19. Elevated D-dimer levels have been reported consistently, with emphasis on progressive increase in their levels, thus indicating a worsening of the condition. Other abnormalities of coagulation tests, such as prolongation of prothrombin time (PT) and activated partial thromboplastin time (APTT) and elevation of fibrin degradation products and severe thrombocytopenia, indicate the possibility of occurrences of disseminated intravascular coagulation (DIC), which needs to be monitored and should undergo early intervention.

Both hospitalized patients and outpatients with COVID-19 are at increased risk of venous thromboembolism. Therefore, early and prolonged pharmacological thromboprophylaxis with low molecular weight heparin is recommended.^18^

Other relevant laboratory parameters include lymphopenia and elevations of PT, D-dimer and lactic dehydrogenase activity, which have already been observed in the initial phase of response to the presence of the virus. With the onset of pneumonia and the consequent hypoxia, there is an increase in transaminase levels increase, which indicates occurrence of significant hepatocellular distress. Increased liver enzyme activity is an important indicator of the severity of the condition. When the most severe stage of hyperinflammation is reached, accompanied by coagulopathy, inflammation markers such as CRP, ferritin, troponin and brain natriuretic peptide (BNP) will be at very high levels, thus characterizing an inflammatory storm.
